# Risk Factors and Predictive Score for Bacteremic Biliary Tract Infections Due to Enterococcus faecalis and Enterococcus faecium: a Multicenter Cohort Study from the PROBAC Project

**DOI:** 10.1128/spectrum.00051-22

**Published:** 2022-06-30

**Authors:** Marco Mussa, Pedro María Martínez Pérez-Crespo, Luis Eduardo Lopez-Cortes, Pilar Retamar-Gentil, Adrián Sousa-Dominguez, Ane Josune Goikoetxea-Aguirre, José María Reguera-Iglesias, Eva León Jiménez, Isabel Fernández-Natal, Carlos Armiñanzas-Castillo, Lucía Boix-Palop, Jordi Cuquet-Pedragosa, Miguel Ángel Morán Rodríguez, Jonathan Fernandez-Suarez, Alfonso del Arco-Jiménez, Alfredo Jóver-Saenz, Alberto Bahamonde-Carrasco, Fátima Galan-Sanchez, Juan Manuel Sánchez-Calvo, Alejandro Smithson-Amat, David Vinuesa-García, Antonio Sánchez-Porto, Inmaculada López-Hernández, Jesús Rodríguez-Baño, the PROBAC REIPI/GEIH-SEIMC/SAEI group

**Affiliations:** a Fondazione IRCCS Ca' Granda Ospedale Maggiore Policlinico, Infectious Diseases Unit, Milan, Italy; b UGC Enfermedades Infecciosas y Microbiología, Hospital Universitario Virgen Macarena, Sevilla, Spain; c Departamento de Medicina, Universidad de Sevilla/IBiS/CSIC, Sevilla, Spain; d Centro de Investigación Biomédica en Red en Enfermedades Infecciosas (CIBERINFEC), Sevilla, Spain; e Hospital Universitario de Vigo, Vigo, Spain; f Hospital de Cruces, Bilbao, Spain; g Hospital Regional de Málaga, Málaga, Spain; h Hospital Universitario de Valme, Sevilla, Spain; i Complejo Asistencial Universitario de León, León, Spain; j Hospital Universitario Marqués de Valdecilla, Santander, Spain; k Hospital Universitari Mútua de Terrasa, Barcelona, Spain; l Hospital General Granollers, Barcelona, Spain; m Hospital Universitario de Burgos, Burgos, Spain; n Hospital Universitario Central de Asturias, Oviedo, Spain; o Hospital Costa del Sol, Marbella, Spain; p Hospital Universitario Arnau de Vilanova, Lleida, Spain; q Hospital de El Bierzo, Ponferrada, Spain; r Hospital Universitario Puerta del Mar, Cadiz, Spain; s Hospital de Jerez de la Frontera, Jerez de la Frontera, Spain; t Hospital de l’Esperit Sant, Santa Coloma de Gramenet, Barcelona, Spain; u Hospital Universitario San Cecilio, Granada, Spain; v Hospital del SAS de La Línea de la Concepción, Cádiz, Spain; Wayne State University

**Keywords:** bloodstream infection, *Enterococcus* spp., biliary tract infection

## Abstract

Biliary-tract bloodstream infections (BT-BSI) caused by Enterococcus faecalis *and* E. faecium are associated with inappropriate empirical treatment and worse outcomes compared to other etiologies. The objective of this study was to investigate the risk factors for enterococcal BT-BSI. Patients with BT-BSI from the PROBAC cohort, including consecutive patients with BSI in 26 Spanish hospitals between October 2016 and March 2017, were selected; episodes caused by E. faecalis or E. faecium and other causes were compared. Independent predictors for enterococci were identified by logistic regression, and a predictive score was developed. Eight hundred fifty episodes of BT-BSI were included; 73 (8.5%) were due to target *Enterococcus* spp. (48 [66%] were E. faecium and 25 [34%] E. faecalis). By multivariate analysis, the variables independently associated with *Enterococcus* spp. were (OR; 95% confidence interval): cholangiocarcinoma (4.48;1.32 to 15.25), hospital acquisition (3.58;2.11 to 6.07), use of carbapenems in the previous month (3.35;1.45 to 7.78), biliary prosthesis (2.19;1.24 to 3.90), and moderate or severe chronic kidney disease (1.55;1.07 to 2.26). The AUC of the model was 0.74 [95% CI0.67 to 0.80]. A score was developed, with 7, 6, 5, 4, and 2 points for these variables, respectively, with a negative predictive value of 95% for a score ≤ 6. A model, including cholangiocarcinoma, biliary prosthesis, hospital acquisition, previous carbapenems, and chronic kidney disease showed moderate prediction ability for enterococcal BT-BSI. Although the score will need to be validated, this information may be useful for deciding empirical therapy in biliary tract infections when bacteremia is suspected.

**IMPORTANCE** Biliary tract infections are frequent, and a significant cause of morbidity and mortality. Bacteremia is common in these infections, particularly in the elderly and patients with cancer. Inappropriate empirical treatment has been associated with increased risk of mortality in bacteremic cholangitis, and the probability of receiving inactive empirical treatment is higher in episodes caused by enterococci. This is because many of the antimicrobial agents recommended in guidelines for biliary tract infections lack activity against these organisms. To the best of our knowledge, this is the first study analyzing the predictive factors for enterococcal BT-BSI and deriving a predictive score.

## INTRODUCTION

Biliary tract infections are frequent, and a significant cause of morbidity and mortality if not adequately treated (1). Bacteremia is common in these infections, particularly in the elderly and patients with cancer (2, 3). Although Gram-negative bacteria are the most frequent cause of these infections, enterococci cause 10 to 23% of bacteremic infections (2, 3). Inappropriate empirical treatment has been associated with increased risk of mortality in bacteremic cholangitis (4, 5), and the probability of receiving inactive empirical treatment is higher in episodes caused by enterococci (4). This is because many of the antimicrobial agents recommended in guidelines for biliary tract infections lack activity against these organisms (6, 7). However, to the best of our knowledge, there is no studies identifying specific risk factors for covering enterococcal etiology in biliary tract infections.

The objectives of this study are to investigate the risk factors for Enterococcus faecalis *and* Enterococcus faecium bacteremic biliary tract infections and to develop a predictive score for this etiology. Our ultimate goal is to provide helpful information to guide empirical therapy in patients with biliary tract infections to improve coverage of enterococci when necessary, while avoiding the ecological impact and the possible side effects of overtreatment. In addition, we provide a detailed description of bacteremic biliary tract infections caused by these organisms.

## RESULTS

Overall, 6313 episodes of bacteremia were included in the PROBAC cohort, and 850 (13.4%) had a biliary tract source. Among them, 73 (8.6%) were caused by the pathogens of interest, of which 48 were E. faecium (66%) and 25 E. faecalis (34%). Seven BT-BSI episodes caused by other enterococcal species (all of them were polymicrobial) were excluded; 5 were caused by *E. gallinarum* and by 2 *E. caselliflavus*.

Among the 73 included episodes of BT-BSI caused by enterococci, 37 (51%) were polymicrobial; the most frequent co-pathogens were Escherichia coli (14; 37.8%), Klebsiella pneumoniae (9; 24.3%), K. oxytoca (4; 10.8%), Citrobacter freundii (2; 5.4%), and Enterobacter cloacae (2; 5.4%).

The main demographic and clinical characteristics of patients with BT-BSI are described in Table 1. Their median age was 76 years; 313 (36.8%) were women and had a median age-adjusted Charlson index of 5. The most frequent comorbidities were cancer (252, 29.6%) and diabetes mellitus (212; 24.0%). Overall, 268 (31.5%) had biliary tract obstruction and 18% a biliary tract prosthesis; 201 (23.6%) had received antimicrobial therapy in the previous month. With regard to the severity of infection, 251 (20.5%) presented with severe sepsis or shock, and 344 (49.5%) had a SOFA score ≥2.

**TABLE 1:** Demographic, epidemiological, and baseline characteristics of bloodstream infections from a biliary tract source, and according to isolation of Enterococcus faecalis and Enterococcus faecium in blood cultures

Variables	All patients *n* =850 (%)	Isolation of E. faecalis *or* E. faecium (*n* = 73)	No isolation - others (*n* = 777)	P value
Demographic				
Median age in yrs (IQR)	76 (65.84)	75 (63-83)	76 (66-84)	0.11
Female sex	313 (36.8)	29 (39.7)	284/775 (36.6)	0.60
Acquisition				
Community acquired	452 (53.2)	22 (30.1)	430 (55.4)	<0.001
Community-onset, health-care related	224 (26.4)	16 (21.9)	208 (26,8)	0.36
Hospital acquired	173 (20.4)	35 (47.9)	138 (17.8)	<0.001
Comorbidities				
Median age-adjusted Charlson comorbidity index (IQR)	5 (3-7)	6 (4-8)	5 (3-7)	0.038
Congestive heart failure	68 (8.0)	4 (5.5)	64 (8.2)	0.50
Hypertension	65 (7.6)	6 (8.2)	59 (7.6)	0.84
Dementia	61 (7.2)	6 (8.2)	55 (7.1)	0.71
Cerebrovascular disease	58 (6.8)	4 (5.5)	54 (6.9)	0.27
Chronic obstructive pulmonary disease	90 (10.6)	8 (11)	82 (10.6)	0.91
Diabetes mellitus	212 (24.9)	18 (24.7)	194 (25)	0.95
Diabetes mellitus with organ damage	43 (5.1)	5 (6.8)	38 (4.9)	0.40
Moderate/severe liver disease	83 (9.8)	14 (19.2)	69 (8.9)	0.005
Chronic kidney disease (stage 4-5)	75 (8.8)	11 (15.1)	64 (8.2)	0.04
Connective tissue disorder	18 (2.1)	2 (2.7)	16 (2.1)	0.66
Peptic ulcer	22 (2.6)	1 (4.5)	21 (2.7)	1.00
Peripheral vascular disease	57 (6.7)	7 (9.6)	60 (6.4)	0.30
Cancer	252 (29.6)	33 (45.2)	219 (28.2)	0.005
Cholangiocarcinoma	15 (1.8)	6 (8.2)	9 (1.2)	<0.001
Pancreatic cancer	13 (1.5)	3 (4.1)	10 (1.3)	0.093
Hematologic cancer	18 (2.1)	2 (2.7)	16 (2.1)	0.69
Urinary obstruction	9 (1.1)	0	9 (1.2)	1.00
Recurrent urinary tract infection	15 (1.8)	3 (4.1)	12 (1.5)	0.11
Biliary tract obstruction	268 (31.5)	27 (37)	241 (31)	0.29
Immunosuppressive therapy	67 (7.9)	12 (16.4)	55 (7.1)	0.005
Neutropenia <500 cells/μL	12 (1.4)	0 (0)	12 (1.5)	0.28
Medical device and procedures				
Biliary tract prosthesis	153 (18)	24 (32.9)	129 (16.6)	0.001
Surgery (30 days before)	48 (5.6)	9 (12.3)	39 (5)	0.010
Biliary surgery	32 (3.8)	7 (9.6)	25 (3.2)	0.006
Parenteral feeding	17 (2)	5 (6.8)	12 (1.5)	0.002
Esophagogastroduodenoscopy	13 (1.5)	2 (2.7)	11 (1.4)	0.30
Endoscopic retrograde cholangiopancreatography	5 (0.6)	2 (2.7)	3 (0.4)	0.061
Other upper gastrointestinal endoscopy	50 (5.9)	12 (16.4)	38 (4.9)	<0.001
Colonoscopy	4 (0,5)	0	4 (0.5)	1.00
Bronchoscopy	34 (4)	8 (11)	26 (3.3)	0.002
Mechanical ventilation	8 (0.9)	4 (5.5)	4 (0.5)	0.003
Previous antimicrobial treatment (30 days before)				
Any antimicrobial	201 (23.6)	30 (41.4)	171 (22)	<0.001
Beta-lactam/beta-lactam inhibitor	115 (13.5)	20 (27.4)	95 (12.2)	<0.001
Carbapenem	32 (3.8)	11 (15.1)	21 (2.7)	<0.001
Third generation cephalosporin	10 (1.2)	9 (1.2)	1 (1.4)	0.87
Severity at presentation				
Sepsis and septic shock	251 (29.5)	28 (38.4)	223 (28.7)	0.084
SOFA ≥ 2	344 (40.5)	37 (50.7)	307 (39.5)	0.080
Pitt score > 3	60 (7.1)	8 (13.3)	52 (6.7)	0.17

Regarding exposure to potential predisposing factors, BT-BSI due to enterococci were more frequently nosocomially acquired, the patients had a higher Charlson index, a higher rate of moderate or severe liver disease, chronic kidney disease (stage 4 or 5) and cholangiocarcinoma, and more frequently had a biliary prosthesis and had undergone previous surgery, an endoscopic procedure and mechanical ventilation; also, they had more frequently received immunosuppressant drugs and antibiotics in the prior month, particularly beta-lactam/beta-lactam inhibitors and carbapenems (Table 2).

**TABLE 2:** Multivariate analysis of risk factors and predictive score for BT-BSI due to Enterococcus faecalis and Enterococcus faecium

Variable	β coefficient	Or (95% CI)	P value	Score
Cholangiocarcinoma	1.501	4.48 (1.32-15.25)	0.01	+7
Hospital acquisition	1.276	3.58 (2.11–6.07)	<0.001	+6
Carbapenem use in the previous mo	1.211	3.35 (1.45–7.78)	0.005	+5
Biliary prosthesis	0.785	2.19 (1.24–3.90)	0.02	+4
Chronic kidney disease stage 4 or 5	0.441	1.55 (1.07–2.26)	0.02	+2

The multivariate analysis selected biliary prosthesis, cholangiocarcinoma, hospital acquisition, use of carbapenem in the previous 30 days and chronic kidney disease stage 4 or 5 as independent risk factors for enterococcal etiology (Table 2). The AUROC of the model for observed mortality was 0.74 (95% CI 0.67 to 0.80), indicating a moderate prediction ability. The NPV, PPV, sensibility and specificity for the different values of the score are showed in Table 3. A score value ≥6 showed a NPV of 95% with a specificity of 80.4%; overall, the number of BT-BSI patients with a score <6 in our cohort were 657 (77.4%) and enterococci was isolated in 33 (5%) of them. On the other side, a score value ≥10 (which were 58 [6.8%] of the BT-BSI population) showed a PPV of ≥32.8% (19 of them had *Enterococcus* spp. in blood cultures). When the score was applied to monomicrobial BSI due to *Enterococcus* spp., the AUC was 0.85 (95% CI 0.80 to 0.91); for monomicrobial *E. facium*, the AUC was 0.87 (95% CI0.81-0.93), and for monomicrobial E. faecalis, 0.75 (95% CI0.60-0.81).

**TABLE 3:** Positive predictive values (PPV), negative predictive values (NPV), sensitivity, and specificity for biliary tract bacteremia caused by Enterococcus faecalis and Enterococcus faecium according to the different values of the PROBAC enterococcal score

Score points	VPP	VPN	Sensitivity	Specificity	No. of episodes	No. of *faecalis* and *faecium* infection
2	15.7	96.5	76.7	61.2	357	56
3	16.7	95.8	72.6	67.8	300	50
4	16.7	95.8	68.5	67.8	300	50
5	20.3	94.9	54.8	79.8	197	40
6	20.8	95.0	54.8	80.4	192	40
7	31.6	93.7	32.9	93.3	76	24
8	31.0	93.7	30.1	93.7	71	22
9	33.3	93.4	28.8	94.6	63	21
10	32.8	93.2	26	95.0	58	19
11	41.9	92.7	17.8	97.7	31	13
12	50.0	92.1	9.6	99.1	14	7
13	46.2	92.0	8.2	99.1	13	6
14	44.4	91.8	5.5	99.4	9	9
15	44.4	91.8	5.5	99.4	9	4
16	50.0	91.7	4.1	99.6	6	3
17	60	91.7	4.1	99.7	5	3
18	100	91.6	2.7	100	2	2
19	100	91.6	2.7	100	2	2
20	100	91.5	1.4	100	1	1
21	100	91.5	1.4	100	1	1
22	100	91.5	1.4	100	1	1

For a strictly descriptive interest, crude comparison of outcome data of enterococcal and other etiologies of BT-BSI are shown in Table 4. Overall, enterococcal BT-BSI in our cohort showed statistically significant higher proportions of inappropriate empirical therapy (43.8% versus 22.8%, *P* = 0.0002), relapses (11.0% versus 4.6%, *P* = 0.02) and secondary infections (4.1% versus 0.9%, *P* = 0.04), and statistically nonsignificant, numerically higher rates of mortality (21.9% versus 14.4%, *P* = 0.08) and persistent bacteremia (5.5% versus 1.8%, *P* = 0.06).

**TABLE 4:** Outcomes of patients with biliary tract bloodstream infections according to isolation of Enterococcus faecalis and Enterococcus faecium

Variables	All patients *n* =850 (%)	Isolation of E. faecalis or E. faecium (*n* = 73)	No isolation others (*n* = 777)	P value
Inappropriate empirical therapy	209 (24.5)	32 (43.8)	177 (22.8)	0.0002
30-day mortality	128 (15.1)	16 (21.9)	112 (14.4)	0.087
Persistent fever	97 (11.4)	11 (15.1)	86 (11.1)	0.30
Persistent bacteremia	18 (2.1)	4 (5.5)	14 (1.8)	0.061
Relapse	44 (5.2)	8 (11.0)	36 (4.6)	0.020
Secondary infection^*a*^	10 (1.2)	3 (4.1)	7 (0.9)	0.047

a Endovascular or orthopedic device infections.

The empirical drugs received by patients with enterococcal BT-BSI were (several patients received combination empirical treatment) piperacillin-tazobactam (30 patients), meropenem (20), linezolid (9), ciprofloxacin or levofloxacin, vancomycin and cephalosporins (7 each), daptomycin and metronidazole (5 each), teicoplanin, amoxicillin-clavulanic acid and imipenem (3), and aztreonam, gentamicin, ampicillin and colistin (1 each).

## DISCUSSION

In this study, the prevalence, features, outcomes, and risk factors for BT-BSI due to E. faecalis or E. faecium were described; the independent risk factors associated with this etiology were cholangiocarcinoma, biliary prosthesis, hospital acquisition, previous use of carbapenem and chronic kidney disease. Also, a predictive score with moderate prediction ability was developed.

Inappropriate empirical therapy has been associated with increased mortality in some studies of BT-BSI, and isolation of enterococci is related to inappropriate therapy, as was the case in this study; therefore, providing information about when these organisms should be covered empirically is relevant (5, 8). Many of the antibiotics recommended in guidelines for biliary tract infections are not active against enterococci (i.e., cephalosporins, aztreonam, and some of the carbapenems in the case of E. faecalis; or all beta-lactams in most of cases of E. faecium). Addition of vancomycin is recommended in all health care-associated infections in the Tokyo and IDSA guideline (6, 7); this would have mean treating with vancomycin 379 (46.8%) of the BT-BSI episodes in our cohort (i.e., all nosocomial and health care-associated cases), of which only 51 (13.4%) had enterococci isolated in blood cultures. The Tokyo guidelines also recommended vancomycin for grade III cases; we did not use that severity scale, but considering those with sepsis or shock as a proxy, 122 episodes with community-acquired BT-BSI who presented with sepsis or shock would have also needed to be treated with vancomycin, of which only 9 (7.4%) were caused by enterococci. Therefore, the sensitivity and specificity of the criteria recommended in the Tokyo guidelines for covering entecococci in our cohort was 38.4% and 71.3%, respectively, with a positive predictive value of 11.2% and negative predictive value of 92.5%.

To the best of our knowledge, this is the first study analyzing the predictive factors for enterococcal BT-BSI and deriving a predictive score. The small number of cases did not allow us to perform an internal validation; although the predictive score developed needs external validation and showed a moderate predictive ability, it may be of help in the decision to cover enterococci in patients with biliary tract infections. The limited discriminative ability of our score may be due to the high rate of polymicrobial BSI cases since it raised significantly when applied to monomicrobial episodes and particularly those caused by E. faecium. In addition, we analyzed E. faecium and E. faecalis together but the risk factor for BSI due to these organisms may not be the same (9).

In patients with a biliary tract sepsis, empirical coverage against Gram-negative bacteria is mandatory in all cases. We think that patients with a score ≤6 and without septic shock may be safely treated empirically without coverage against enterococci. On the other hand, coverage for *Enterococcus* spp. should be considered in patients with a score ≥10. In patients with a score between 7 and 9, the need to cover enterococci should be evaluated considering the severity of infection and other generic risk factors for enterococcal bacteremia regardless the source beyond the specific variables included in the score (i.e., immunosuppression, other types of cancer, previous use of other broad-spectrum antimicrobials) (9).

In our analysis, the presence of cholangiocarcinoma increased the risk for enterococci by 4.5-fold. In a prospective observational study, including 173 episodes of bacteremic cholangitis, E. faecium was the third most frequent bacteria isolated on blood cultures in patients with solid tumors (and mainly hepato-biliary-pancreatic cancer) (2). Also, bile duct colonization by E. faecium has been shown to be associated with infection in patients with perihiliar cholangiocarcinoma (10, 11). The association of biliary stents with *Enterococcus* spp. infection has been previously shown (12, 13); *Enterococcus* spp. bacteremia in cholangitis episodes is more frequent when a biliary endoprosthesis is present (12). Chronic kidney disease has been described as a risk factor for enterococcal BSI in several studies, even if not focusing on BT-BSI (12, 13). Finally, the use of carbapenems is a well-recognized risk factors for enterococcal bacteremia, and particularly for E. faecium (14–16).

This study has several limitations. First, the number of enterococcal BT-BSI included might have limited the statistical power to find some risk factors. Second, we did not collect specific data on the different type of biliary tract infections. Third, the results are not applicable to BT infections without bacteremia. And fourth, the predictive score was not externally validated. Some strengths include the prospective nature of the study and the multicenter representation.

In conclusion, we found some specific risk factors for enteroccoccal BT-BSI and derived a predictive score that might be useful for guiding decisions about coverage of these organisms, pending its validation in external cohorts.

## MATERIALS AND METHODS

### Design, setting, and patients.

This is a *post hoc* analysis of the PROBAC project, a prospective, observational, multicenter cohort study, including consecutive patients with bloodstream infections (BSI) admitted to 26 Spanish hospitals from October 2016 to March 2017; the design and inclusion criteria have been described elsewhere (8, 17).

For this sub-analysis, patients in the PROBAC cohort for whom the biliary tract was considered to be the source of BSI (BT-BSI) were eligible. The biliary tract was considered the source of the BSI on the basis of clinical and radiologic data, including right upper quadrant pain, jaundice, elevated bilirubin levels and radiological signs of cholecystitis or biliary tract obstruction; the microorganisms isolated in bile or surgical samples were checked when available but only for confirmation of the source of bacteremia (patients without bacteremia were not included). Both polymicrobial and monomicrobial bloodstream infections were included. Blood cultures were obtained, processed, and interpreted in accordance with standard recommendations from the Spanish Society of Infectious Diseases and Clinical Microbiology (18).

### Variables and definition.

BT-BSI were classified into those caused by E. faecalis or E. faecium and those caused by other pathogens. Episodes caused by species of enterococci different from E. faecalis or E. faecium were excluded from the analyses because of their very low numbers and potentially different epidemiological and clinical implications. Despite not all species of enterococci were analyzed, from now on we will refer to enterococci or *Enterococcus* spp. for the ease of understanding.

The variables collected included: demographics, underlying conditions and their severity, exposure to invasive procedures and devices during the previous week, type of acquisition (community, health care-associated or nosocomial), acute severity of disease, immunosuppressive therapy and previous antimicrobial use. The severity of underlying conditions was assessed using the age-adjusted Charlson comorbidity index (19). Neutropenia was defined as moderate (<500 neutrophils/μL) and severe (<100 neutrophils/μL). Liver diseases was classified as moderate or severe when the patient presented a B or C class according to Child-Pugh classification (20). Presentation with sepsis or shock was considered according to Sepsis 3 criteria (21, 22). The Sequential Organ Failure Assessment (SOFA) score (23) at presentation and Pitt score (24) 1 day before were also measured. Chronic kidney disease was defined moderate to severe according to the Charlson Comorbidities index parameter (19). Infections were classified as hospital acquired (HA), health care associated (HCA) or community-acquired accordingly to Friedman et al. (25). Immunosuppressive therapy included cancer chemotherapy or radiotherapy, typical immunosuppressants, and steroids if dosing used was > 10 mg/day of prednisone or equivalent for more than 3 weeks.

Outcome variables included inappropriate empirical treatment (i.e., lack of use of *in vitro* active drugs before the susceptibility of blood pathogens isolated was known), 30-day mortality, relapse (reappearance of bacteremia after documentation of negative blood cultures or clinical improvement and completion of active antimicrobial therapy), persistent fever (longer than 72 h in patients treated with an *in vitro* active antimicrobial regimen), persistent bacteremia (isolation of the same bacteria in follow-up blood cultures after 72 h of active antimicrobial regimen), and secondary endovascular or orthopedic devices infection.

### Statistical analysis.

To analyze the risk factors for enterococcal etiology, exposure to the different variables were compared between patients with and without isolation of enterococci in blood cultures using the Chi-square test and Fisher exact test for categorical variables, and the T-Student test and Mann-Whitney U for continuous variables, as appropriate. Stratified analyses were performed whenever necessary to understand the correlation among variables.

Among the possible variables associated with *Enterococcus* spp., those clinically meaningful and with a univariate *P* value <0.1 were included in a multivariate logistic regression model. The variables selection was performed using a manual stepwise backward process, including potential interactions. The predictive ability of the final models for observed data were evaluated by calculating their area under the receiver operating characteristic curve (AUROC) with 95% confidence interval (CI).

A predictive score was developed using the final multivariate model. The value of the predictors included in the score was calculated by dividing each beta coefficient by half of the smallest one and rounding to the nearest unit. The positive predictive value (PPV), negative predictive value (NPV), sensitivity and specificity of the score were calculated. Statistical analysis was carried out with the Statistical Package for the Social Sciences version 18.0 (SPSS Inc., Chicago, IL, USA).

### Ethical approval.

The PROBAC project was approved by the Ethics Committee of Hospital Universitario Virgen Macarena. Approval was also obtained at each participating center according to local requirements. The need to obtain written informed consent was waived because of the observational nature of the study.
